# BioActive packaging materials based on pectin/curdlan gum and Pau d’Arco extract with pro-health properties as a solution for food industry

**DOI:** 10.1038/s41598-026-56446-0

**Published:** 2026-06-11

**Authors:** Renata Dobrucka, Marcin Szymański, Lukas Vapenka, Elżbieta Studzińska-Sroka, Magdalena Paczkowska-Walendowska, Judyta Cielecka-Piontek, Małgorzata Lasik-Kurdyś, Małgorzata Gumienna

**Affiliations:** 1 Department of Non-Food Products Quality and Packaging Development, Institute of Quality Science, Poznań University of Economics and Business, al. Niepodległości 10, 61-875 Poznan, Poland; 2 Center for Advanced Technologies, Adam Mickiewicz University in Poznań, Uniwersytetu Poznańskiego 10, 61-614 Poznan, Poland; 3 Department Food Preservation, University of Chemistry and Technology Prague, Technicka 5, Prague 6, Czech Republic; 4 Department of Pharmacognosy and Biomaterials, Poznan University of Medical Sciences, Rokietnicka 3, 60-806 Poznan, Poland; 5 Faculty of Food Science and Nutrition, Poznań University of Life Sciences, Wojska Polskiego 31, 60-624 Poznan, Poland

**Keywords:** Active packaging materials, Pectin, Curdlan gum, D’Arco extract, Bioinspired materials, Sustainability

## Abstract

In the present study, bioactive packaging materials based on polysaccharides were developed and qualified by the WHO/FAO Expert Committee as safe food additives. Pau d’Arco extract was incorporated due to its rich bioactive composition and strong antioxidant properties. The addition of the extract conferred significant antioxidant activity to the polymer films. The colour of the films varied significantly (p < 0.05) depending on the extract concentration, which itself exhibited a dark colour. The highest barrier performance in terms of water vapour transmission rate (WVTR) and oxygen transmission rate (OTR) was observed for the sample with the highest extract content (2.5F). Changes in WVTR were associated with the presence of polyphenols and flavonoids in the extract. Furthermore, the films displayed concentration-dependent antibacterial effects against *E. coli*, with higher extract levels leading to longer inhibition times, indicating enhanced antimicrobial activity. Spearman’s correlation confirmed that antioxidant activity parameters and the total polyphenol and flavonoid content were fully correlated with OTR and WVTR (R = 1), as well as with other measured properties, demonstrating the multifunctional potential of these bioactive films.

## Introduction

Food packaging industry is under pressure to comply with more sustainable practices, in which the circular economy plays an essential role. This concept has become a central focus for many governments aiming to promote sustainable economic development. The food packaging industry is a particularly intriguing *circular economy* context due to the health and safety requirements associated with food^1^. One of the problems experienced in the food industry is the migration of substances from food contact materials. It is estimated that there are more than 16 000 known chemicals in plastics, encompassing both substances used in production and substances added inadvertently^2^. The chemicals in plastics, which are mostly non-covalently bound to the polymer, can be released from plastic products and contaminate the natural and human environment. One of these is Bisphenol A (BPA). BPA belongs to a group of compounds consisting of two phenolic rings, it is a class of synthetic compounds widely used in the production of polycarbonate plastics^3^. According to the literature, approximately 10 million tonnes of bisphenol A are produced annually worldwide to meet the demands of the polymer industry. In 2023 alone, the global BPA market size reached 9.22 million metric tonnes, a compound annual growth rate (CAGR) of 3.0%, and is projected to reach 12.03 million metric tonnes by 2032^4^.

BPA washes out of materials, with the result that products are becoming less preferred by consumers due to safety concerns, especially products that come into contact with baby food^5^. Unfortunately, as scientific studies show, BPA has negative effects on human health^6^. BPA can interfere with thyroid hormone signalling at various target sites by inhibiting the sodium-iodide symporter (NIS), altering the expression of related genes, etc. Due to BPA’s high potential to interfere with metabolism, development, and impair neurological and reproductive functions^7^. In response to the research-proven negative effects of BPA, a regulation came into force on 20 January 2025 banning the use of BPA and its salts in the production of materials and articles intended to come into contact with food. BPA is also present in the environment. BPA can contaminate surface and groundwater through industrial discharges, urban runoff and leaching from landfills and plastic waste. It is a major pollutant in aquatic environments. Its concentrations in water sources can range from low nanogram to microgram per litre^8^. Therefore, these are difficult times for the packaging industry, especially food packaging in the context of both a circular economy and the search for solutions that do not pose migration risks for people and the environment^9,10^.

Nowadays, there is a growing global awareness of the environmental issues associated with the use of synthetic and non-degradable packaging, which has increased interest in bio-based packaging made from natural and biodegradable polymers. Strict regulations, rising consumer demand for environmentally friendly products, and technological advancements are driving the development of such materials. As a result, a gradual transformation is underway, during which industries and supply chains are adapting to more sustainable packaging solutions. Biopolymer-based materials are gaining significant attention due to their positive environmental impact and consumer safety. They are characterized by biodegradability, non-toxicity, and biocompatibility, which can significantly reduce the environmental burden associated with the production and use of conventional packaging^9–11^. Polysaccharide-based materials, in particular, are gaining prominence. Polysaccharides, among the most abundant biopolymers in nature, are long-chain macromolecules formed by linking monosaccharides via glycosidic bonds^12^. Many regulatory authorities have evaluated them as safe and suitable for direct contact with food in the form of films, supporting their practical application in the food industry. Additionally, polysaccharides can enhance immune function and promote gut health, increasing their appeal as food-preserving agents^11^. However, it is important to note that films made solely from single polysaccharides have certain limitations, such as low water vapor barrier properties, brittleness, and limited elongation at break, which can reduce their effectiveness in food protection^9,10^. Studies have shown that incorporating active components into such films can significantly improve their mechanical strength and reduce water vapor permeability^13,14^. Therefore, current research focuses on optimizing film formulations and combining polysaccharides with other natural additives or biopolymers to develop more durable and functional packaging materials.

In the present study, packaging materials were developed based on polysaccharides of plant origin, namely apple pectin (PA) and curdlan gum (CG). These materials were deliberately selected from polysaccharides that have been previously evaluated and classified by the WHO/FAO Expert Committee as food additives, ensuring their suitability for applications related to food contact. Pectin is an anionic, amorphous and non-toxic polymer naturally occurring in the cell wall of plants, mainly in various skins and flesh of fruits and vegetables. Most commercial pectin comes from apple pomace (methylation, 24%) and citrus peels (methylation, 74%)^15^. In the food industry, pectin is beginning to replace fat and serve as a functional ingredient that promotes health in addition to its conventional uses as a gelling agent, thickener and stabiliser^16^. Pectin is a heteropolysaccharide derived from the cell walls of higher plants. As such, pectin has been classified as GRAS by the FDA. The favourable biocompatibility, edibility and physical properties of pectin make it a suitable material to produce edible food packaging films^17^. Curdlan gum is a new type of exopolysaccharide synthesised by *Alcaligenes faecalis* and the genus Agrobacterium. Curdlan, a bacterial extracellular natural linear polysaccharide composed of glucose through β-1,3 glycosidic bonds^18^. Due to its gelling properties, curdlan is an excellent candidate for use as an edible film for food packaging. However, its lack of bioactive properties also limits its use in functional packaging. Consequently, curdlan gum combined with bioactive substances to achieve specific functions has become an important direction in functional packaging applications^19^. There are few reports in the literature on combinations of pectin with curdlan gum. Furthermore, it is difficult to find literature reports on the use of Pau d’Arco extract to obtain innovative packaging biomaterials.

Pau d’Arco (LaPacho, *Tabebuia impetiginosa*) is a plant belonging to the Bignoniaceae family that is mainly found in the Amazon rainforest and other tropical regions of Central and Latin America^20^. The internal bark of *Tabebuia avellanedae* has been used as a folk medicine to treat malaria, fever, trypanosomiasis, fungal and bacterial infections and gastric disorders^21,22^. In recent years, several studies have shown that extracts or compounds isolated from *Tabebuia impetiginosa* exhibit a wide range of pharmacological activities, such as anti-obesity, antifungal, antipyretic, antioxidant, anti-inflammatory and anticancer activities^23^. The most characteristic active constituents found in Pau d’Arco cortex are lapachol and β-lapachone. Lapachol inhibits tumour cell proliferation, while β-lapachone shows strong toxicity in mouse and human cells^20^. Many other compounds are also present in Pau d’Arco cortex—including phenolic glycosides, lignans, isocoumarins, benzenoids, alkaloids, flavonoids, quinones, tannins and mineral salts, as well as coenzyme Q^21,22^. Due to the presence of bioactive compounds and the documented health-promoting properties of Pau d’Arco bark, this extract was selected for film preparation. The primary aim of this study was to develop a bioactive, polysaccharide-based packaging material incorporating Pau d’Arco extract, characterized by high antioxidant and antibacterial activity. Additionally, the study sought to obtain an environmentally friendly alternative to petroleum-based packaging materials, suitable for food industry applications and offering active protective functions.

## Materials and methods

### Microorganisms

For antimicrobial activity 5 test strains were selected: *Limosilactobacillus oris* DSM 4864, *Limosilactobacillus fermentum* DSM 20,055 and *Lacticaseibacillus paracasei* DSM 20,312 from DSMZ – German Collection of Microorganisms and Cell Cultures GmbH (Germany); *Bacillus subtilis* NCIMB 11,871 from National Collection of Industrial and Marine Bacteria, (United Kingdom) and *Escherichia coli* from our own Faculty Microbial Collection (Poznan University of Life Sciences, Poland). For the analysis a liquid monocultures of the tested bacteria were prepared. *Escherichia coli* and *Bacillus subtilis* were cultivated for 24 h at 37 °C using the appropriate growth medium MacConkey (Sigma-Aldrich, Germany) for *E. coli* and nutrient broth (Merck, Germany) for *B. subtilis. L. oris, L. fermentum* and *L. paracasei* were cultivated for 48 h using MRS broth medium (Merck, Germany). The concentration of inoculum was adjusted to 0.5 McFarland standard.

### Plant material

The plant material used in this study, Pau d’Arco (the bark of a plant), was purchased from the NANGA Przemysław Figura store in Blękwit, Poland. As the material was commercially available, no special permits were required for its acquisition. Prior to extraction, the bark was ground into a fine powder using a laboratory mill.

### Preparation of extract

The film preparation started with the preparation of Pau d’Arco extract. 50.0 g of Pau d’Arco cortex was placed in a round-bottom flask, and 250 mL of 50% ethanol was added. The mixture was heated at the boiling point of the solvent under a reflux condenser for 2 h. The extract was then filtered and the bark was returned for a second extraction under the same conditions. This was repeated three times. The filtrates were combined, and the ethanol was evaporated with part of the water to a volume of 50 ml, yielding a 50% ethanol extract in water at a concentration of 1 g/ml. The ethanol and part of the water were evaporated using an evaporator at a temperature of 80 °C under reduced pressure.

### Preparation of film samples

Film-forming solutions were prepared by combining apple pectin (PA), glycerine, curdlan gum (CG), and water in a 1:1:1:100 ratio. Appropriate amounts of Pau d’Arco extract were then added according to the desired concentration. The prepared solutions were stirred magnetically at 500 rpm for 65 min at 95 °C. The resulting films were dried at 22 °C for 72 h to obtain thin, uniform films. The applied process conditions were selected to ensure proper film formation while maintaining the functional properties of both the polysaccharide matrix and the bioactive component. The samples were labeled according to the concentration of Pau d’Arco extract, as follows: 0F (control, no extract), 0.5F (0.5 wt.% extract), 1.5F (1.5 wt.% extract), and 2.5F (2.5 wt.% extract).

### GC–MS analysis of extract

2.0 g of milled Pau d’Arco cortex was placed in a 25-ml round-bottom flask, 10 ml of ethanol was added, heated for 10 min at boiling point on a heating bowl, then cooled and filtered through a 0.45 µm syringe filter. Chromatographic studies were performed on a GC–MS chromatograph (SCION TQ, BRUKER). 1 µl of the solution was injected onto the column. The chromatograph was equipped with a VF-5 ms Crawford Scientific silica column. The electron energy was 70 eV, and the ion source was at 200 °C. Helium was used as the carrier gas at a flow rate 1 ml/min. Temperature program: Enable Coolant at 50.0 °C, Coolant Timeout 20.00 min, Stabilization Time 0.50 min.; Temperature 60.0 °C, Hold 3.00 min., Total 3.00 min.; Temperature 280.0 °C, Rate 10.0 °C/min., Hold 35.00 min., Total 60.00 min. The identification of compounds was based on a comparison of their retention time as well as mass spectra with NIST standards.

The estimated detection and quantification limits for the analyzed compounds in EI-GC–MS (SCAN) mode were approximately 0.1–5 ng and 0.3–15 ng per column, respectively.

### Chemical analysis the extract using high-performance liquid chromatography (HPLC)

Using high-performance liquid chromatography, the presence and content of 4-hydroxybenzoic acid was detected and determined in water extract of Pau d’Arco cortex. The analysis was performed using the HPLC system (Thermo Scientific Dionex UltiMate 3000 UHPLC) according to the method described by Paczkowska-Walendowska et al.^24^. Process parameters were: detection wavelength: 265 nm,mobile phase flow: 1 ml/min; column temperature: 40 °C; mobile phase: A- 0.1% formic acid, B-acetonitrile, according to the gradient: 0–35 min: 2–20% B; 35–55 min: 20–70% B; 55 min: 2% B; 55–60 min: 2% B. Analysis procedure: solution of standard substance was prepared by weighing and dissolving aliquots in HPLC-grade methanol (4-hydroxybenzoic acid 0.1 mg/ml). The obtained solutions were filtered through a membrane filter (0.22 µl), and injections were made (50 µl to 5 µl). The standard curve was determined, and the method was validated by determining the parameters, i.e., linearity, indirect and direct precision, limit of quantification (LOQ), and limit of detection (LOD).

### Dissolution studies

Dissolution studies of prepared films were performed using the modified method described by Paczkowska-Walendowska et al.^24^. Briefly, 40 ml of water was added to the beaker and a 4 cm^2^ films (0.5F, 1.5F, 2.5F) were placed inside; the experiment was conducted at room temperature (n = 3). At set time points (0.5 h, 1.0 h, 2.0 h, 4.0 h, 6.0 h, and 24 h), a sample of the fluid was taken, filtered through a 0.22 μm nylon membrane, and the concentration of 4-hydroxybenzoic acid was determined using the previously developed HPLC method.

### Determination of total flavonoid content (TFC)

The analysis was performed following the method used by Studzińska-Sroka et al.^25,26^. In short, 100.0 µl of the tested Pau d’Arco extract (100 mg dried weight of plant material (DW)/ml), films (10 mg of film/ml) or quercetin (7.8–125 μg/ml) was mixed with 100.0 µl of 2% solution of aluminium chloride. In dark conditions, the plate was shaken for 10 min at room temperature at 500 rpm, and the absorbance was read at 415 nm using the microplate reader (Multiskan GO 1510, Thermo Fisher Scientific, Vantaa, Finland). The blank contained a sample and water or methanol instead of 2% water or methanol (for quercetin) solution of aluminium chloride. The results were the average from n = 6 measurements (for extract) or n = 3 measurements (for films), was estimated using the standard calibration curve (y = 19.32x − 0.0128, R^2^ = 0.9993), and were expressed as milligram of quercetin equivalent (QE)/g DW plant material or mg QE/g film ± standard deviation (SD).

### Determination of total phenolic content (TPC)

The analysis was performed following the method used by Studzińska-Sroka et al.^25,26^. To 25 µl of the Pau d’Arco extract (10 mg DW/ml), films (10 mg of film/ml) or gallic acid solution (in the concentration range 6.25–100 μg/ml), 200 µl of distilled water, 15 µl of Folin-Ciocalteu reagent and 60 µl of 20% sodium carbonate solution were added. In dark conditions, the plate was shaken at room temperature at 500 rpm and incubated for 30 min at room temperature. The absorbance was read at 760 nm using the microplate reader (Multiskan GO 1510, Thermo Fisher Scientific, Vantaa, Finland). The results were the average from n = 4 measurements, was estimated using the standard calibration curve (y = 5.7959x − 0.0017, R^2^ = 0.9991), and expressed as milligram of gallic acid equivalents (GAE)/g DW plant material or mg GAE/g film ± standard deviation (SD).

### DPPH assay

A previously described DPPH analysis was used to determine the antiradical activity^25,26^. Briefly, 25.0 μl of the Pau d’Arco extract at 0.3125 -10 mg DW/ml, the film solution (10 mg of film/ml), or Trolox was combined with 175.0 μl of DPPH solution (3.9 mg/50 ml of methanol). The mixture was shaken in the dark at 500 rpm for 30 min at room temperature. Absorbance was measured at 517 nm using a plate reader (Multiskan GO 1510, Thermo Fisher Scientific, Vantaa, Finland). Each test was conducted in duplicate, and the final result was calculated as the average of six measurements (n = 6) for the extract, and of four measurements (n = 4) for the films. The control contained 25.0 μl of water and 175.0 μl of DPPH solution. The blank was the mixture of 25 μl of distillate water and 175 μl of methanol. The results were expressed as IC_50_ (mg/ml) for Pau d’Arco extract and as mg Trolox equivalent (TE)/g of film (concentrations of Trolox ranged from 0.0078125 to 0.1 mg/ml).

### ABTS assay

A previously described ABTS analysis was used to determine the antiradical activity with slight modifications^27^. Briefly, 10.0 μl of Pau d’Arco extract (0.313 – 10.0 mg/ml) or film solutions (10 mg of film/ml) or Trolox were mixed with 200.0 μl of ABTS^•+^ solution (7 mM ABTS^•+^ prepared in 2.45 mM potassium persulfate solution and diluted with water to absorbance value ~ 0.77). The mixture was shaken in the dark at 500 rpm for 30 min at room temperature. Absorbance was measured at 734 nm using a plate reader (Multiskan GO 1510, Thermo Fisher Scientific, Vantaa, Finland). The control contained 10.0 μl of water and 200.0 μl of ABTS^•+^ solution. The blank was the mixture of 210 μl distillate water. The results were expressed as IC_50_ (mg/ml) for Pau d’Arco extract and as mg TE/g of the film (concentrations of Trolox ranged from 0.2 to 0.025 mg/ml).

### CUPRAC assay

The CUPRAC (CUPric Reducing Antioxidant Capacity) analysis was conducted using the procedure described previously^25,26^. The CUPRAC reagent was prepared by mixing equal volumes of neocuproine solution (7.5 mM), copper (II) chloride solution (10 mM), and ammonium acetate buffer (1 M, pH = 7.0). Then, 50 μl of the tested Pau d’Arco extract (0.15625 – 10.0 mg DW/ml) or the film solution (10 mg of film/ml) and 150 μl of the CUPRAC reagent were mixed. The mixture was shaken in the dark at 500 rpm for 30 min in a dark place at room temperature. Absorbance was measured at 450 nm using a plate reader (Multiskan GO 1510, Thermo Fisher Scientific, Vantaa, Finland). Blank was an attempt to replace 50.0 μl of the tested extract with 50.0 μl of water. The assay was repeated twice, and the result represent the average of four determinations (n = 4). The results were expressed as IC_0.5_ (mg/ml) for Pau d’Arco extract and as mg TE/g of film (concentrations of Trolox ranged from 0.003125 to 0.2 mg/ml).

### FRAP assay

The FRAP analysis was conducted using the procedure described previously^28^ with slight modifications. The FRAP reagent was prepared by 25 ml of the acetate buffer pH = 3.6 and 2.5 ml of 10 mM TPTZ in 40 mM HCl with 2.5 ml of 20 mM FeCl_3_ × 6H_2_O aqueous solution. Then, 20 μl of Pau d’Arco extract (0.15625—10 mg DW/ml) or the film solution (10 mg of film/ml) and 180 μl of the FRAP reagent were mixed. The mixture was shaken in the dark at 500 rpm for 30 min in a dark place at room temperature. Absorbance was measured at 593 nm using a plate reader (Multiskan GO 1510, Thermo Fisher Scientific, Vantaa, Finland). Blank was an attempt to replace 20.0 μl of the tested extract with 20.0 μl of water. The assay was repeated twice, and the result represent the average of four determinations (n = 6). The results were expressed as IC_0.5_ (mg/ml) for Pau d’Arco extract and as mg TE/g of film or DW (concentrations of Trolox ranged from 0.0015625 to 0.2 mg/ml).

### Determination of Fe^2+^ chelating activity

The binding of Fe^2+^ by the extracts was effectuated according to Studzińska-Sroka et al.^29^, with some modifications. Briefly, 200.0 μl of Pau d’Arco extract (0.06125 – 0.5 g DW/ml) or the film solution (10 mg of film/ml), and 10.0 μl of FeCl_2_·4H_2_O (1 mM) were mixed and pre-incubated in 96 well plates at room temperature for 10 min, shaking 500 at rpm. Afterwards, 10 μl of ferrozine (2.5 mM) was added and incubated at room temperature for 30 min. The absorbance of the iron (II)-ferrozine complex was measured at 562 nm. The assay was repeated twice, and the result represent the average of three determinations (n = 3). The results were expressed as IC_50_ (g/ml) for Pau d’Arco extract and as mg EDTA equivalent/g of the film (concentrations of EDTA ranged from 0.01 to 0.08 mg/ml).

### Anti-hyaluronidase activity

The anti-hyaluronidase activity was performed according to the previously described method^25,26^. Briefly, 25.0 µl of incubation buffer (acetate buffer pH 4.5, with 77 mM NaCl and 1 mg/ml albumin), 25.0 µl of enzyme solution (30 U/ml), 10.0 µl of the tested extract (10 mg DW/ml) or film solutions (10 mg of film/ml), and 15.0 µl of acetate buffer were mixed in the well. After 15 min of incubation (37 °C) with shaking at 500 rpm, 25.0 µl of hyaluronic acid (HA) solution (0.3 mg/ml) was added and incubated for 45 min with shaking (37 °C). After this time, 200.0 µl of CTAB solution (2.5%) in 2% sodium hydroxide was added. After 10 min of incubation without shaking (at room temperature), the absorbance (λ = 600 nm) was measured using a plate reader (Multiskan GO 1510, Thermo Fisher Scientific, Vantaa, Finland). The composition of blanks was previously described^30^. The determination was repeated twice and calculated from four results (n = 9). The results are expressed as a percentage of inhibition ± SD.

### Antibacterial activity

The evaluation of antimicrobial activity of the bioactive extracts and films was performed using electrical impedance changes method. The principle of this method is to analyze the changes of electrical impedance caused by metabolic activity of incubated microorganisms in the liquid growth media^31–33^. During the growth of microorganisms, molecules with high molecular weight (e.g. protein, carbohydrates, lipids), electrically neutral or low ionized are catabolized by bacteria to ionized metabolic products. In turn, smaller and charged particles are formed, which contribute directly to changes in environmental impedance. The higher the bacterial activity, the more dynamic changes in impedance are detected. The graphic image of these changes (expressed in % in relation to the initial value) is a curve similar to the course of the microorganism growth curve. The parameter used to assess the amount and activity of bacteria is the impedance detection time (IDT). It indicates a moment of distinct increase of impedance. In the experiment a 5% threshold value was settled. The impedance detection time (IDT) is the time when the threshold value (5%) is crossed. The shorter is the IDT the higher is the number and activity of the tested bacteria. This IDT parameter was applied for comparative analysis of inhibition effect of the tested extract and films. The measurements were performed in the Automated Microbiological Growth Analyzer (BacTrac 4100, Sy-Lab, Austria) using 10 cm^3^ BacTrac measuring tubes, equipped with four electrodes. Film analysis: the tested films were sterilized before analysis by exposure to UV light for 30 min. Next, 5 cm^2^ of the film (0F as control; 0.5F; 1.5F and 2.5F) and 0.1 cm^3^ of the bacteria inoculum and were placed in 10 cm^3^ of the growth medium (appropriate for used bacteria) in the BacTrac measuring tubes. Then the test tubes were incubated in the BacTrac 4100 incubator at 37 °C for 48 h and changes of electrical impedance in the growth medium were registered.

Extracts analysis: the tested extract were placed in BacTrac measuring tests tubes with growth medium (appropriate for used bacteria), to reach 5, 10 and 20% concentration. Next 0.1 cm^3^ of the bacteria inoculum was introduced to the medium and the test tubes were incubated in the BacTrac 4100 incubator at 37 °C for 48 h and changes of electrical impedance in the growth medium were registered.

### FTIR analysis

FTIR analysis was carried out with the same apparatus and method as in the previous studies^9,10,34^.

### Colour analysis

The colour test was carried out using an EnviSense NR60CP colourimeter as in previous papers^9,10,34^.

### Density analysis

The film density ρs was calculated according to the following equation:

$$\uprho {\mathrm{s}} = \frac{{\mathrm{m}}}{{{\mathrm{A}} \times \updelta }}$$

where:

m - dry mass of the film after conditioning (g),

A - surface area of the film 2 × 2 (cm^2^),

δ - film thickness (cm),

ρs - is the density of the film (g/cm^3^).

### Swelling index

The swelling index of the film was tested using a modified weight method as described^35^. Each film sample (2 cm × 2 cm) was pre-weighed (SI) and then immersed in 30 ml of distilled water at 25 °C for 1 min (SI1), 30 min (SI30) and 1 h (SI60). After drying the film surface with absorbent paper, the films were finally reweighed (Ws). The swelling index was calculated using the following equation:

$${\mathrm{SI}} = \frac{{{\mathrm{Ws}} - {\mathrm{Wi}}}}{{{\mathrm{Wi}}}} \times 100$$

### Opacity analysis

Opacity values of HP-based films were recorded by measuring the absorbance of the films at 600 nm, divided by the film thickness (mm), using a UV/visible spectrophotometer (SmartSpec 3000 Bio-Rad, Segrate, Milan, Italy), according to the method previously described by. Four strips of each film were cut (1 cm × 4 cm) and placed in the quartz cuvette of the spectrophotometer; air was considered as a reference blank.

### Moisture content

The moisture content of the film samples was analysed according to the following method. Film samples (3 cm × 3 cm) were placed on aluminium dishes and dried on a weighing machine at 105 °C to constant weight. Moisture content was assessed by calculating the difference between their initial (Wi) and final (Wk) weights (before and after drying), using the following equation:

$${\mathrm{Moisture}}\;{\mathrm{content}} = \frac{{{\mathrm{Wi}} - {\mathrm{Wd}}}}{{{\mathrm{Wi}}}} \times 100$$

### Mechanical tests

Strength testing (elongation at break (EB), and tensile strength (TS).) was carried out as in previous work^9,10,34^.

### Barrier studies

Water vapor transmission rate (WVTR) was determined by gravimetrically method according to standard DIN 53 122 at 23 °C and relative humidity of 50%. Permeance of the film to oxygen gas ($P_{{O_{2} }}$) was determined using OxTran 2/20 MH measuring system (MOCON Inc., USA) according to standard ASTM D3985—17 at 23 °C and relative humidity of 50%. Aluminum self-adhesive mask for reduction of sample tested area (5.73 cm^2^ instead of standard 50.00 cm^2^) was used for measurement of permeance $P_{{O_{2} }}$ with extended measurement range. Due to the different thicknesses of the films, the transmission rate and permeance values were referred to a uniform thickness of 100 µm for relative comparison of material barrier properties.

### Migration test

Migration tests were performed in principle according to the series of standards EN 1186. Testing conditions 10 days at 40 °C were performed according to the requirements of Regulation 10/2011 for covering storage at room temperature or below, including when packaged under hot-fill conditions, and/or heating up to a temperature 100 °C for a maximum time 15 min. Overall migration into neutral food simulant—10% ethanol (w/w), into evaporable substitution of fatty food simulant—95% ethanol (w/w) and dry food simulant—modified polyphenylene oxide (MPPO, Tenax® TA, 60–80 mesh) was tested. The tests were carried out in 30 ml glass vials equipped with a plastic screw cap with a Teflon septum and an aluminum foil insert. A foil sample with a total area of 3.14 cm^2^ was placed in the lid. Amounts: 3.75 ml (10% or 95% ethanol) or 0.12 g (MPPO) was placed in the vial. Subsequently, the vial was closed with a lid with the sample and turned bottom up. Under the given period for migration test, the level of overall migration was determined gravimetrically for 10% ethanol and 95% ethanol by weighing of non-volatile residue on microbalance XPR36 (Mettler Toledo, Switzerland) after evaporation of simulant from vials used for migration test at 90 °C for 10% ethanol and at 75 °C for 95% ethanol in drying chamber with natural convection. Determination of overall migration into dry food simulant was processed by three-step extraction of Tenax® by shaking (1 min) by diethyl ether (1^st^ 2.4 ml, 2^nd^ and 3^rd^ 3.6 ml) in glass vial used for migration test with decanting and collecting of each diethyl ether extract through filter into new 15 ml glass vial. Non-volatile residue for weighing on microbalance was obtained by evaporation of diethyl ether from filtered a collected extracts in 15 ml vial on dry bath sample concentrator MINI-100N (Miulab, China) at temperature 30 °C and constant flow of nitrogen above the diethyl ether extracts level. All laboratory consumables and chemicals used above were purchased from Sigma Aldrich (Merck, Germany) and P-LAB (Czech Republic).

### Image of the obtained films

Scanning microscopy and stereo microscopy studies were carried out on the same models and using the same technique as in previous work^9,10,34^.

### Statistical analysis

In order to verify the correlation between the samples, the values of the parameters (TS, EB, WVTR, OTR, opacity, colour parameters, density, SM, SI1, SI30, SI60, Total polyphenols, antioxidants, migration, oxygen permeability) were tabulated and Spearman’s rank correlation test was performed. Correlation coefficients are significant at p < 0.05. Calculations were performed using STATISTICA 13. Variables were assumed to be: uncorrelated for R = 0; weakly correlated for 0 < R < 0.5; strongly correlated for 0.5 ≤ R < 0.7; very strongly correlated for 0.7 ≤ R < 0.9; almost fully correlated for 0.9 ≤ R < 1; fully correlated for R = 1. Cluster analysis was also performed. It was calculated using STATISTICA ver. 13.

## Results and discussion

### GC analysis of Pau d’Arco extract

**Fig. 1:**
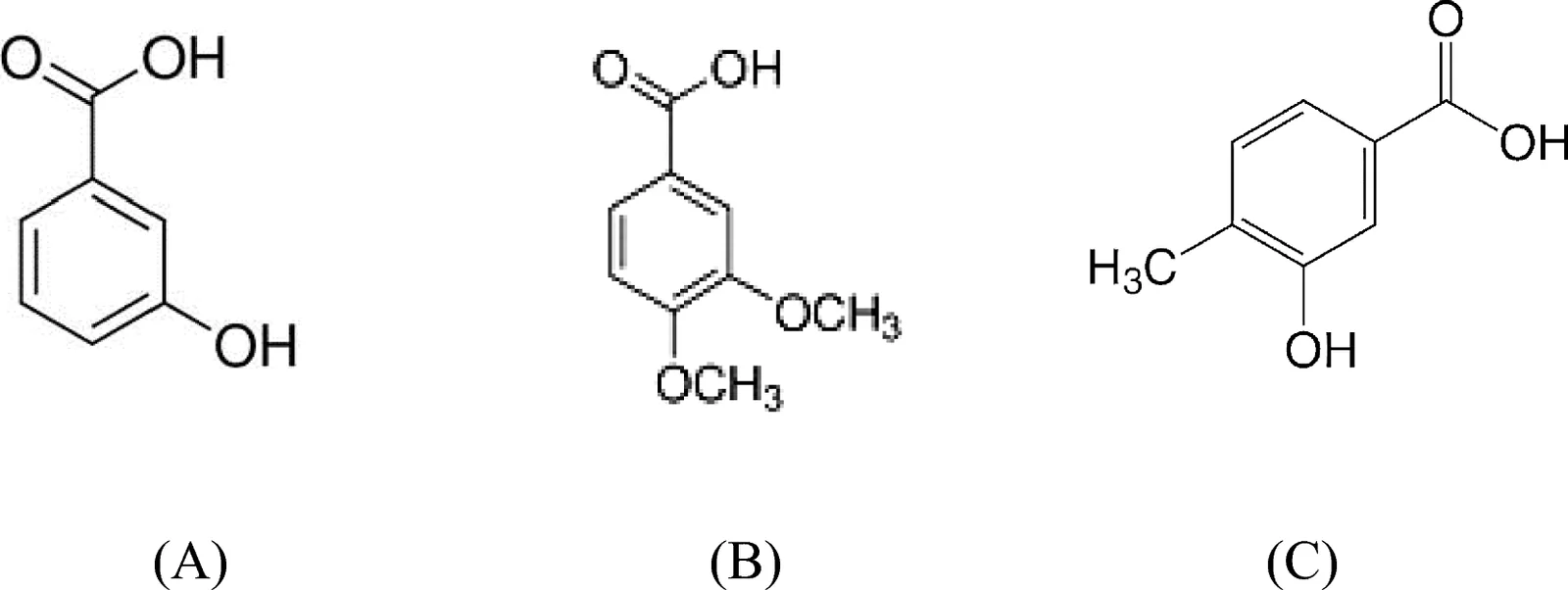
Chemical structure of selected compounds contained in Pau d’Arco cortex extract (**A**) m-salicylic acid, (**B**) veratric acid and (**C**) 3,4-cresotic acid.

GC–MS analysis of the ethanolic Pau d’Arco cortex extract allowed the identification of 22 compounds (Table 1). The most abundant in the extract were m-Salicylic acid (18.60%), 3,4-Cresotic acid (15.00%), Veratric acid (14.25%) (Fig. 1).

**Table 1:** GC–MS results of Pau d’Arco cortex extract.

**Peak RT** **(min)**	**Area**	**Quantity** **(%)**	**Compound name**	**Formula**
8.848	5.86E + 06	0.92	Methyl 6-oxoheptanoate	C_8_H_14_O_3_
10.073	4.20E + 06	0.66	(E)-Cinnamaldehyde	C_9_H_8_O
10.206	1.69E + 07	2.66	2-Methylcumarone	C_9_H_8_O
11.178	3.48E + 07	5.49	(2E,4E)-7-Hydroxy-6-methyl-2,4-octadienoic acid	C_9_H_14_O_3_
11.376	7.33E + 06	1.16	5-(1-Hydroxy-2-propenyl)-2,2-dimethylcyclohexanone	C_11_H_18_O_2_
11.808	4.56E + 06	0.72	D-Melezitose	C_18_H_32_O_16_
12.135	6.31E + 06	1.00	Ascaridole epoxide	C_10_H_16_O_3_
**14.017**	**9.50E + 07**	**15.00**	**3,4-Cresotic acid**	**C**_**8**_**H**_**8**_**O**_**3**_
14.222	4.37E + 07	6.90	Limonen-6-ol, pivalate	C_15_H_24_O_2_
14.680	2.74E + 07	4.32	2’-hexyl-[1,1’-Bicyclopropyl]-2-octanoic acid methyl ester	C_21_H_38_O_2_
**15.145**	**1.18E + 08**	**18.60**	**m-Salicylic acid**	**C**_**7**_**H**_**6**_**O**_**3**_
15.472	3.17E + 07	5.00	4-Methoxy-3-(methoxymethyl)phenol	C_9_H_12_O_3_
15.771	2.53E + 07	4.00	Antiarol	C_9_H_12_O_4_
15.891	2.10E + 07	3.31	Geranyl isovalerate	C_15_H_26_O_2_
**16.158**	**9.03E + 07**	**14.25**	**Veratric acid**	**C**_**9**_**H**_**10**_**O**_**4**_
16.798	2.31E + 07	3.64	3a-[(E)-2-Methoxyethenyl]octahydro-1H-inden-1-one	C_12_H_18_O_2_
17.034	1.78E + 07	2.82	2-(4-Hydroxy-3-methoxyphenyl)ethyl 3-O-(6-deoxyhexopyranosyl)-4-O-[(2E)-3-(4-hydroxy-3-methoxyphenyl)-2-propenoyl]hexopyranoside	C_31_H_40_O_15_
19.555	3.12E + 07	4.93	(-)-6-Hydroxymellein	C_10_H_10_O_4_
21.101	9.12E + 06	1.44	(2E,15Z)-14-Methyl-2,15-octadecadien-1-ol	C_19_H_36_O
21.240	8.68E + 06	1.37	Ethyl iso-allocholate	C_26_H_44_O_5_
24.717	1.75E + 06	0.28	Cholesta-5,7,9(11)-trien-3-ol acetate	C_29_H_44_O_2_
25.585	2.06E + 06	0.32	Gibberellic acid	C_19_H_22_O_6_
		98.79		

Bold value indicates statistically significant.

The biological activity of 3,4-cresotic acid is not widely reported in the scientific literature. Available information on the direct biological activity of 3,4-cresotic acid is limited, with most studies focusing on its role in bacterial metabolic processes and on the properties of other derivatives of this compound^36–38^. Although less well known than its o-salicylic isomer, m-salicylic acid shows interesting biological properties with potential applications. It exhibits properties such as antioxidant, potentially anti-inflammatory, antibacterial and keratolytic. The presence of a hydroxyl and carboxyl group makes m-salicylic acid a potential ligand for metal ions, which may be useful in chelation therapy. However, there are no specific studies confirming this activity for m-salicylic acid. The scientific literature does not provide conclusive evidence of its activity. Primary in vitro antimicrobial activity of veratric acid derivatives was evaluated against microorganisms, including Gram-positive and Gram-negative bacteria and fungi. Some of the veratric acid derivatives showed significant in vitro antimicrobial activity. A QSAR study used to find correlations between various physicochemical parameters of veratric acid derivatives and their antimicrobial activity showed the importance of topological parameters in describing antimicrobial activity^39^. Veratric acid derived from plants and fruits, exhibits antimicrobial, anti-inflammatory and other important therapeutic activities^40^. Veratric acid, one of the main benzoic acids isolated from vegetables and fruits, has been found to exhibit anti-inflammatory activity^41^. Due to such wide-ranging health-promoting properties of Pau d’Arco cortex extract, it was this extract that was used to obtain films.

### Analysis of Pau d’Arco cortex extract (HPLC, TFC)

**Fig. 2:**
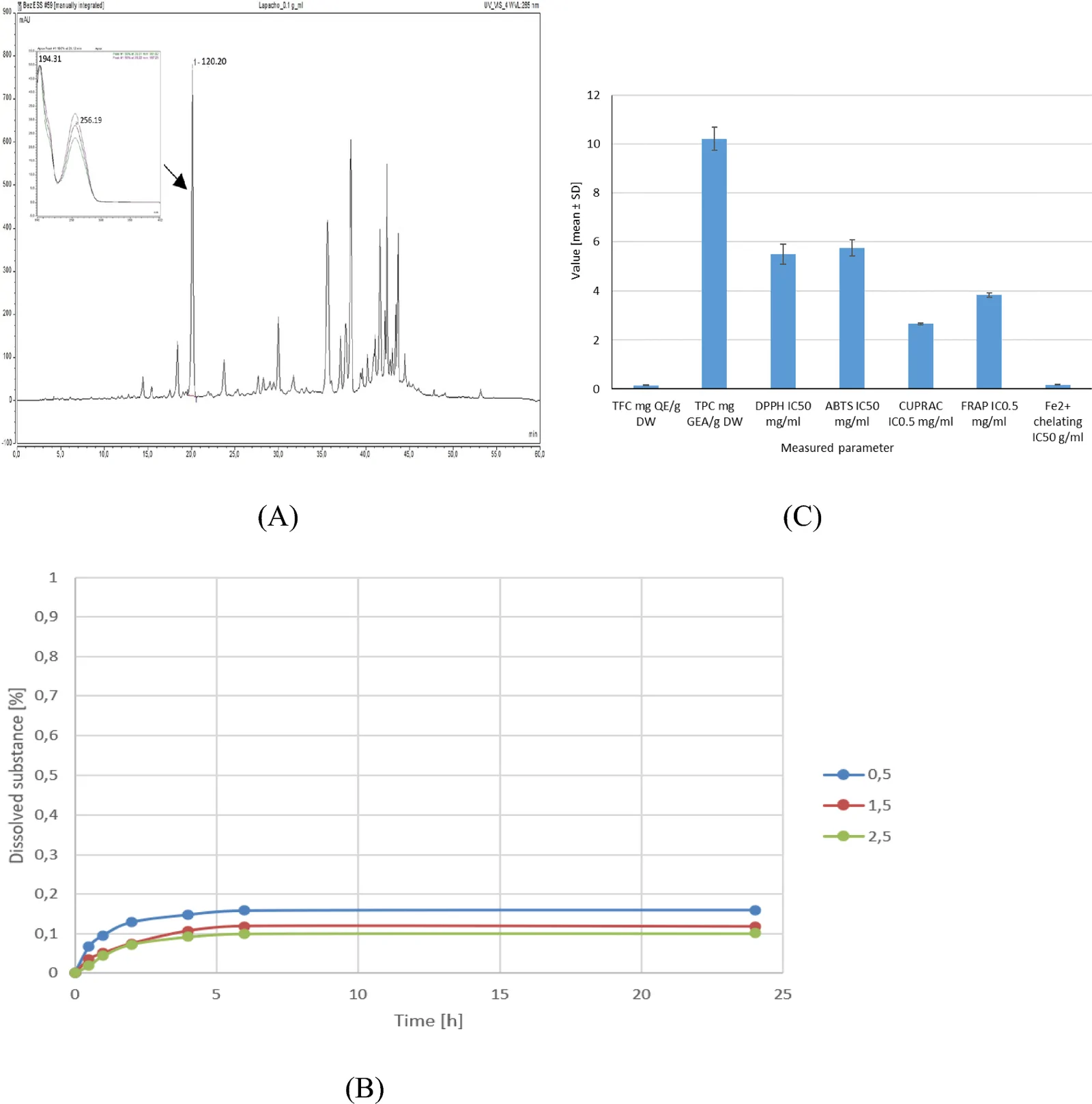
HPLC analysis **(A)**, dissolution studies **(B)** and phenolic compounds content **(C)** (total phenol content – TPC, and total flavonoid content -TFC), and antioxidant potential (DPPH, ABTS, FRAP, CUPRAC, and Fe^2+^ chelating assays) of Pau d’Arco extract.

HPLC analysis of the Pau d’Arco extract confirmed that one of its major compounds is 4-hydroxybenzoic acid (Rt = 20.12 min.) (Fig. 2A), and its content in the extract was 1.23 mg/g DW. The literature search indicated that 4-hydroxybenzoic acid has been previously identified in numerous other plants^42^. Literature reports indicate that this phenolic acid has antioxidant activity, which may explain the results obtained in our study. This compound also has antimicrobial and antimutagenic properties^43^. Furthermore, the release of 4-hydroxybenzoic acid from the films was analyzed. Water-based tests indicated only a minimal (< 1%) release of the acid from the matrix. Based on these results, it can be assumed that the compounds present in the films are unlikely to leach into products with a nearly neutral pH (Fig. 2B).

Phenolic compounds are a large class of plant secondary metabolites widely distributed in various higher plants. Research conducted over recent decades has provided a growing body of evidence of the high biological activity of plants rich in polyphenolic compounds, including their anti-inflammatory, anticancer, antimicrobial, and, above all, antioxidant properties^44^. A high polyphenol content often correlates with free radical scavenging properties, which play a crucial role in mitigating oxidative stress—a process responsible for damaging biological macromolecules such as DNA. Consequently, oxidative stress may lead to cell aging, cardiovascular diseases, and mutagenic changes^45^. Additionally, the antioxidant properties of polyphenols have applications in the food and pharmaceutical industries, helping to extend product shelf life by slowing oxidation processes triggered by pro-oxidants^46^. Thus, given the important role of polyphenols in protecting against oxidative stress, an evaluation of their content in Pau d’Arco cortex extract was undertaken. Moreover, the well-documented high antioxidant potential of flavonoids^47^ provided a basis for analysing their total content in the studied extract. The results obtained indicate that Pau d’Arco extract is characterized by a relatively low polyphenol content and that the flavonoid content in the aqueous extract is negligible (Fig. 2C).

### Antioxidant activity

**Fig. 3:**
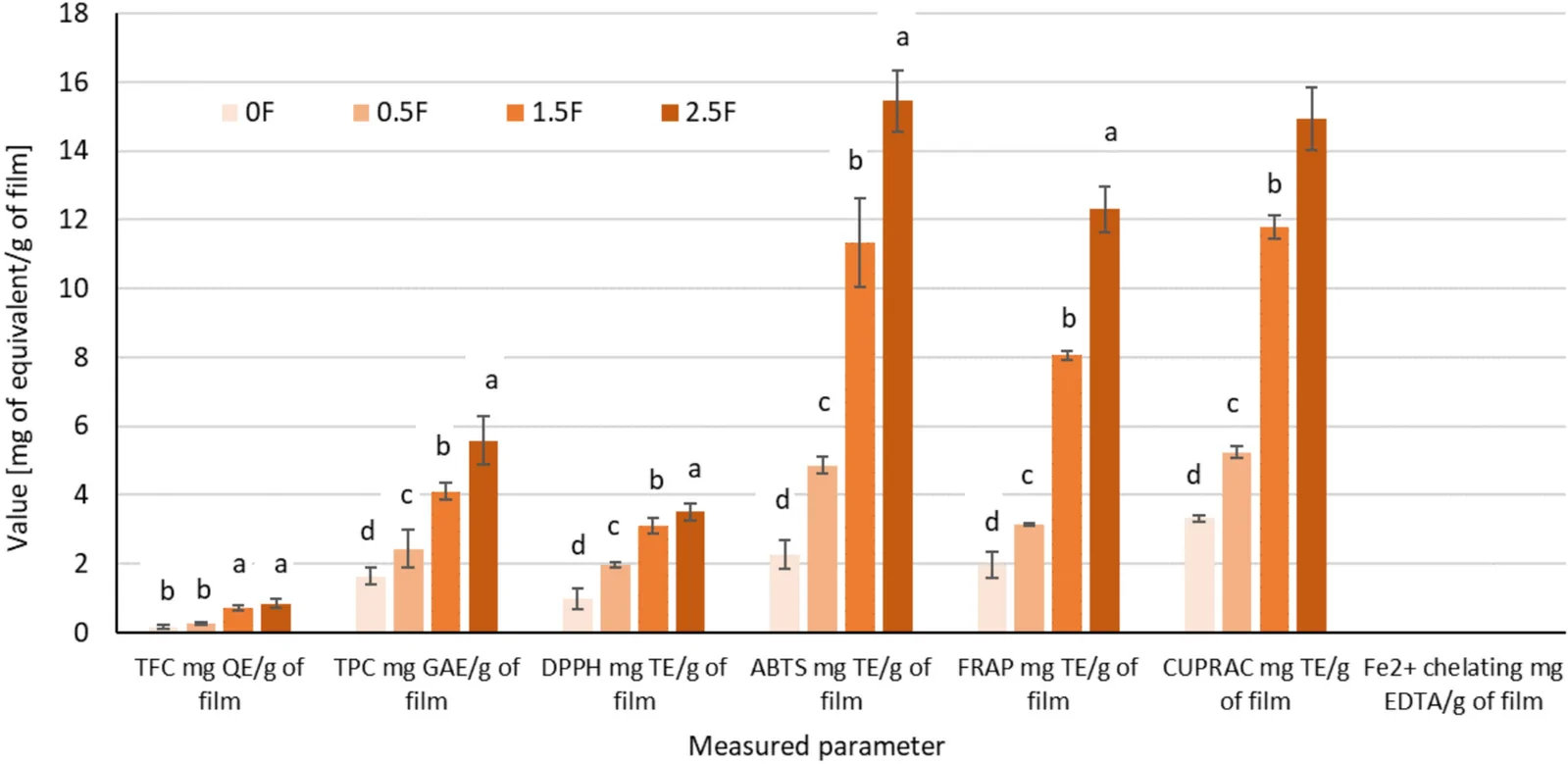
Antioxidant potential (DPPH, ABTS, FRAP, CUPRAC, and Fe^2+^ chelating assays) and phenolic compounds (total phenol content—TPC, and total flavonoid content—TFC) of Pau d’Arco films, examined at 10 mg of film/ml; na—not active.

Various analytical methods were used to evaluate the antioxidant activity in a comprehensive manner. Using a single test could lead to incomplete characterization of antioxidant properties since different methods operate under distinct analytical conditions, such as pH and reaction medium^45^. This approach allowed for a more thorough evaluation of the antioxidant potential of Pau d’Arco extract. In vitro, biological activity studies demonstrated the moderate antioxidant potential of the extract, reflected in its comparable ability to scavenge DPPH^•^ and ABTS^+•^ radicals. The antioxidant effect was further confirmed by the CUPRAC and FRAP assays, which showed the extract’s capacity to reduce transition metal ions Cu^2+^ and Fe^3+^. The relevance of these methods stems from the fact that antioxidants act as reducing agents, neutralizing reactive oxygen species (ROS) through electron donation^48^. However, the analysis of Fe^3+^ ion chelating ability revealed that Pau d’Arco extract has only minimal capacity to bind these ions. The ability to chelate transition metal ions, especially Fe^2+^ and Fe^3+^, may play a crucial role in reducing the intensity of radical-forming reactions and thus mitigating oxidative stress^49^. Further research was carried out on films fortified with Pau d’Arco extract. The results showed that increasing the extract content in the films significantly affected both the total phenolic content (TPC) and the total flavonoid content (TFC) per gram of film. In addition, higher extract concentrations resulted in increased antioxidant activity of the prepared samples. Previous studies have also shown that the addition of Pau d’Arco extract to films increases the biological activity of the films, including antioxidant activity^50^. Films containing Pau d’Arco extract, at the concentrations tested, did not show any iron chelating capacity, which is consistent with the results obtained for the pure extract, indicating a very low level of this activity. It should also be noted that the studies carried out showed that films without the addition of extract, regardless of the method used, showed no antioxidant activity. This justifies the addition of a natural substance with antioxidant activity to the film. The inhibitory activity of Pau d’Arco extract and extract-enriched films against hyaluronidase was assessed to provide complementary information on the biological functionality of the developed materials. The results showed that the tested extract weakly inhibited this enzyme, which is responsible for the degradation of hyaluronic acid in tissues. However, hyaluronidase activity can also serve as a marker of anti-inflammatory potential, as low-molecular-weight hyaluronic acid participates in inflammatory processes^51^. The extract’s low anti-inflammatory potential was similarly observed in films with increasing extract concentrations, as the growing hyaluronic acid content led to diminished enzyme inhibition. On the other hand, the findings suggest that components of the film matrix exhibit anti-hyaluronidase potential. Thus, while the addition of Pau d’Arco extract slightly reduces this activity, it enhances the films with antioxidant properties (Fig. 3).

These results demonstrate the high application potential of the obtained films. The use of films with antioxidant capacity can significantly extend their functional lifetime and, in addition, prolong and protect the packaged food or other oxidation-sensitive product, which can result in a reduction in the use of synthetic food additives.

### Antimicrobial activity evaluation

**Fig. 4:**
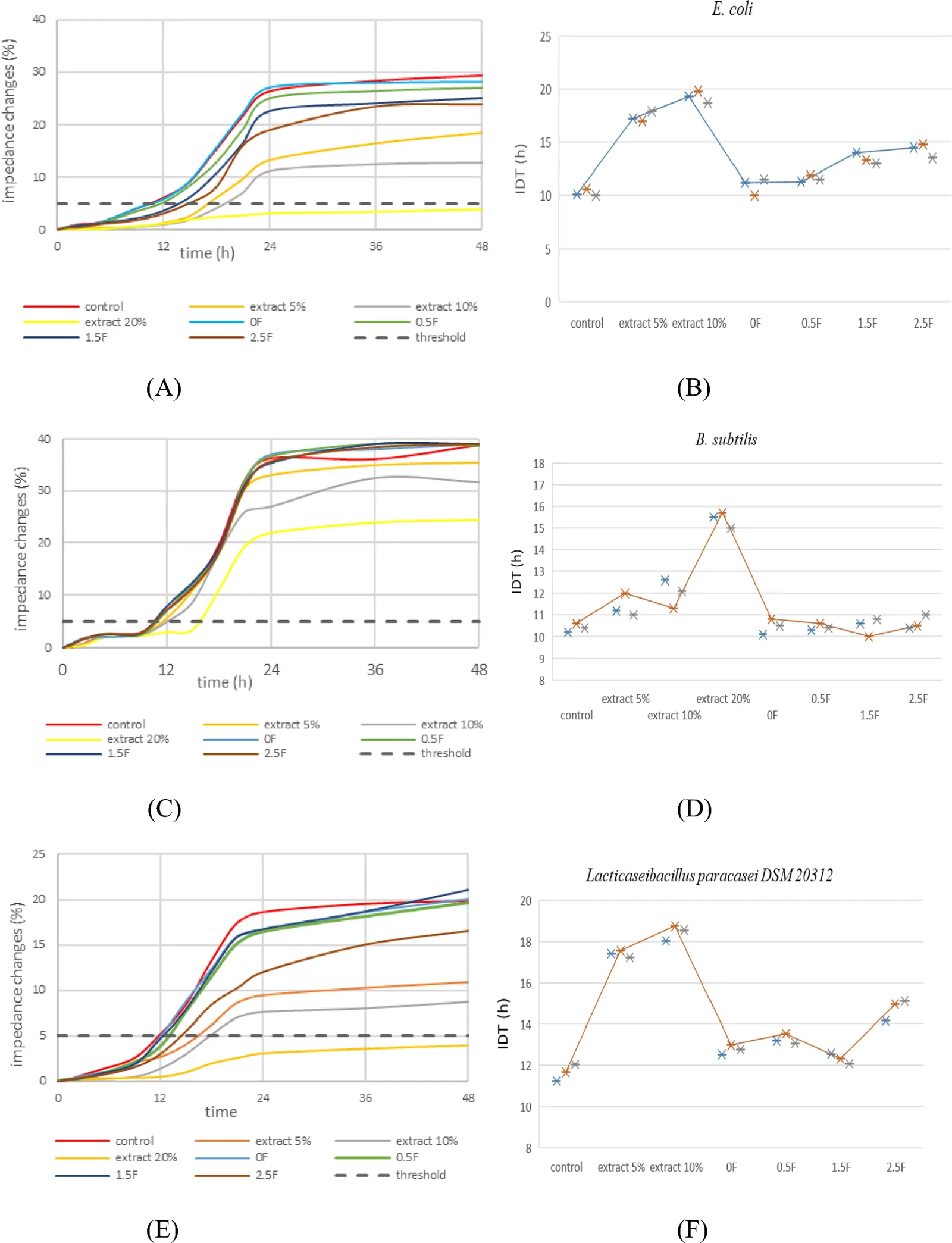

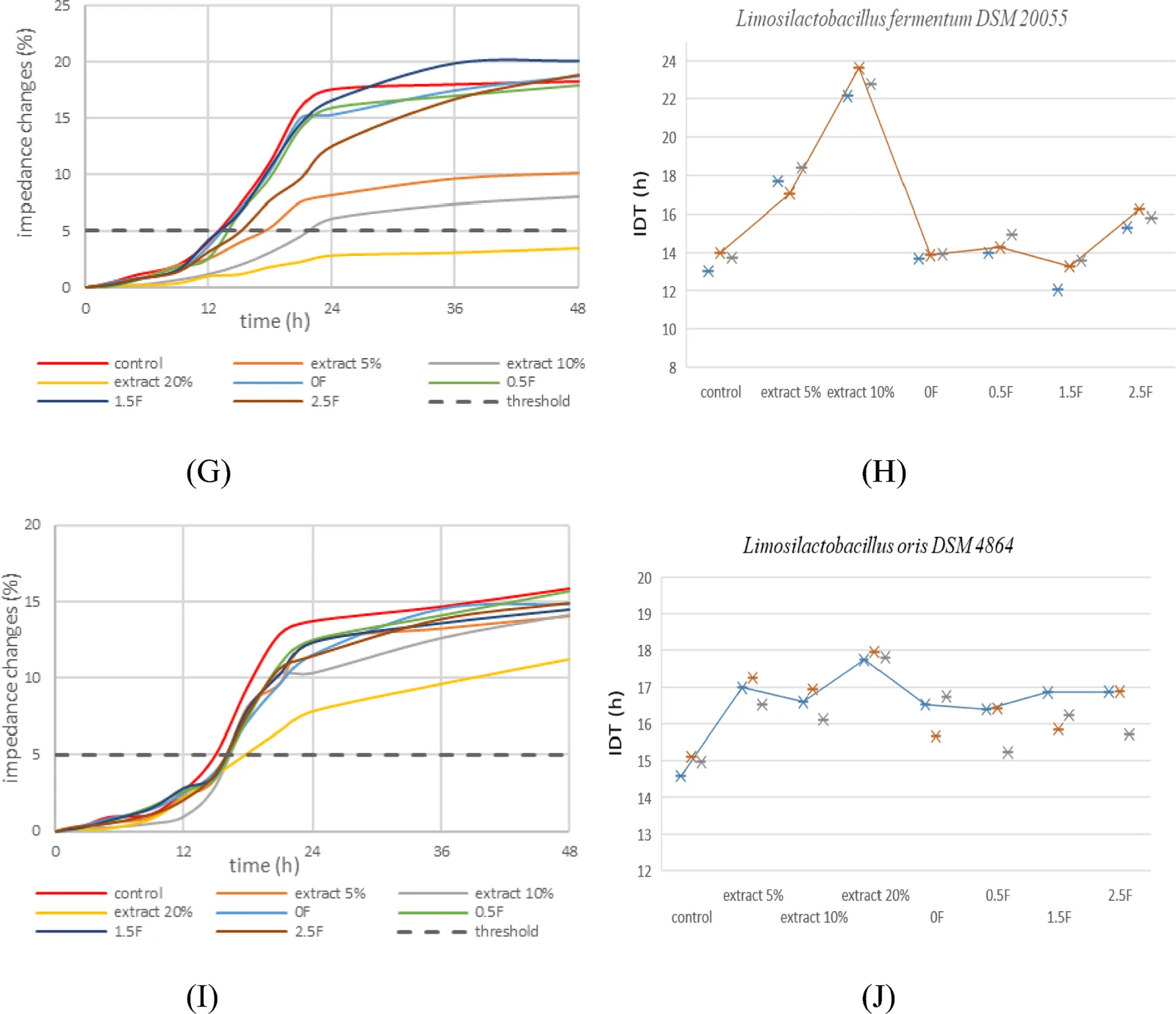
Impedance changes (%) in the growth medium during incubation of selected bacterial strains at 37 °C: *E.coli* **(A)**, *B. subtilis* **(C)**, *L.* *paracasei DSM 20,312* **(E)**, *L. fermentum DSM 20,055***(G)**, *L. oris* DSM 4864, **(I)** and impedance detection time (h) registered at 5% of impedance changes during incubation of *E.coli* **(B)**, *B. subtilis* **(D)**, *L.* *paracasei DSM* 20,312 **(F)**, *L. fermentum DSM 20,055* **(H)**, *L. oris* DSM 4864 **(J)**.

The use of food preservatives based on synthetic chemicals can be dangerous to human health and reduce the food quality^52^. Therefore, the demand for natural antimicrobial alternatives for food products is a priority of consumers. Thus, antibacterial packaging materials will play a very important role, which can show both a bactericidal and bacteriostatic effect in case of bacteria contamination. That is why in this work the antimicrobial activity of the tested extract and all the formed films was assessed. In the performed research the technique of electrical impedance changes measurements was applied as a very sensitive technique detecting bacterial vitality at the level of their metabolic activity. Analyzing the changes in electrical impedance during the growth of tested bacteria in media containing the extract and the tested films, a different course of the curves was observed (Fig. 4). In the impedance changes diagrams a 5% detection threshold was marked to facilitate the interpretation of the results. The differentiation of the course of impedance changes curves indicates different sensitivity of the bacteria to the extract and films. The most sensitive were *E. coli, Limosilactobacillus fermentum* and *Lacticasebacillus parracasei*. The highest tested extract concentration (20%) completely stopped the growth of these strains. Registered impedance changes did not exceed the designated 5%threshold, which can be interpreted as a bactericidal concentration. However, lower extract concentrations: 10 and 5%, showed also a significant inhibitory effect, which can be classified as bacteriostatic (Fig. 4A, E, G). In turn, in the tested films, the concentration of the extract was lower and additionally bioactive substances were blocked in the film matrix, therefore the inhibition effect was significantly weaker. The intensity of the inhibiting effect can be more accurately analyzed using the parameter of impedance detection time (IDT). The higher is the IDT value, the stronger is the antagonistic effect on the tested strain. It means the weakening of bacterial activity (extending the lag-phase), compared to control (variant without an inhibitory factor). It has to be pointed, that IDT values can only be determined for curves in which impedance changes exceeding 5% were registered. For the strains *E. coli*, *Limosilactobacillus fermentum*, and *Lacticasebacillus paracasei*, the parameter could not be recorded for the 20% extract, as the impedance changes were minimal. Lower extract concentrations: 5 and 10% showed a significant inhibitory effect on the bacteria—IDT was significantly extended (Fig. 4). Also, the antagonistic effect of the studied films was observed for the most sensitive strains, i.e. *E. coli, Limosilactobacillus fermentum* and *Lacticasebacillus paracasei*. For *E. coli*, statistically significant differences in the inhibition effect of the tested films were demonstrated. It was correlated with the increase in the concentration of extract in film (as the concentration increased, IDT was extended, indicating higher antibacterial effect). For the *Limosilactobacillus fermentum* and *Lacticasebacillus paracasei* strains, a significant inhibition was recorded only with film 2.5 (Fig. 4). On the other hand, *Bacillus subtilis* and *Limosilactobacillus oris* turned out to be microorganisms resistant to the presence of bioactive ingredients from our films. Both the course of the impedance curves and the IDT parameter did not differ significantly from the results of the control sample (Fig. 4C, D, I, J). The sensitivity of *E. coli* bacteria is a very promising result of the obtained tests. It is a strain whose presence in food is strongly unfavorable due to the causing of infections and spoiling food products. Food dedicated films containing natural substances that significantly inhibit the growth of such adverse microorganisms are therefore very desirable. The clear sensitivity to the bioactive films was also demonstrated by *Limosilactobacillus fermentum* and *Lacticasebacillus paracasei* bacteria. The antimicrobial nature of the studied films can act as microbial stabilization factor in packing fermented foods. Inhibition of bacterial growth can facilitate microbiological control of such products, it can affect, for example, alleviating the parameters of their pasteurization, inhibiting the fermentation process, acting like a natural preservative. Such antibacterial properties of packaging materials clearly affect the safety and quality of food, reducing contaminations and increasing the microbiological stability of packed food. The fact that the bioactive ingredients of the proposed films are plant origin definitely increases their application and market value. Such eco-friendly active packaging systems are part of the strategy of sustainable development, Plant-Based food and modern waste management. Figure 4 presents impedance changes (%) in the growth environment during incubation of selected bacterial strains at 37 °C : *E.coli* (A), *B. subtilis* (C), *L.* *paracasei DSM 20,312* (E), *L. fermentum DSM 20,055* (G), *L. oris* DSM 4864, (I) and impedance detection time (h) registered during incubation of selected bacterial strains at 37 °C on the level of 5% of impedance changes: *E.coli* (B), *B. subtilis* (D), *L.* *paracasei DSM* 20,312 (F), *L. fermentum DSM 20,055* (H), *L. oris* DSM 4864 (J).

### FTIR analysis of films

**Fig. 5:**
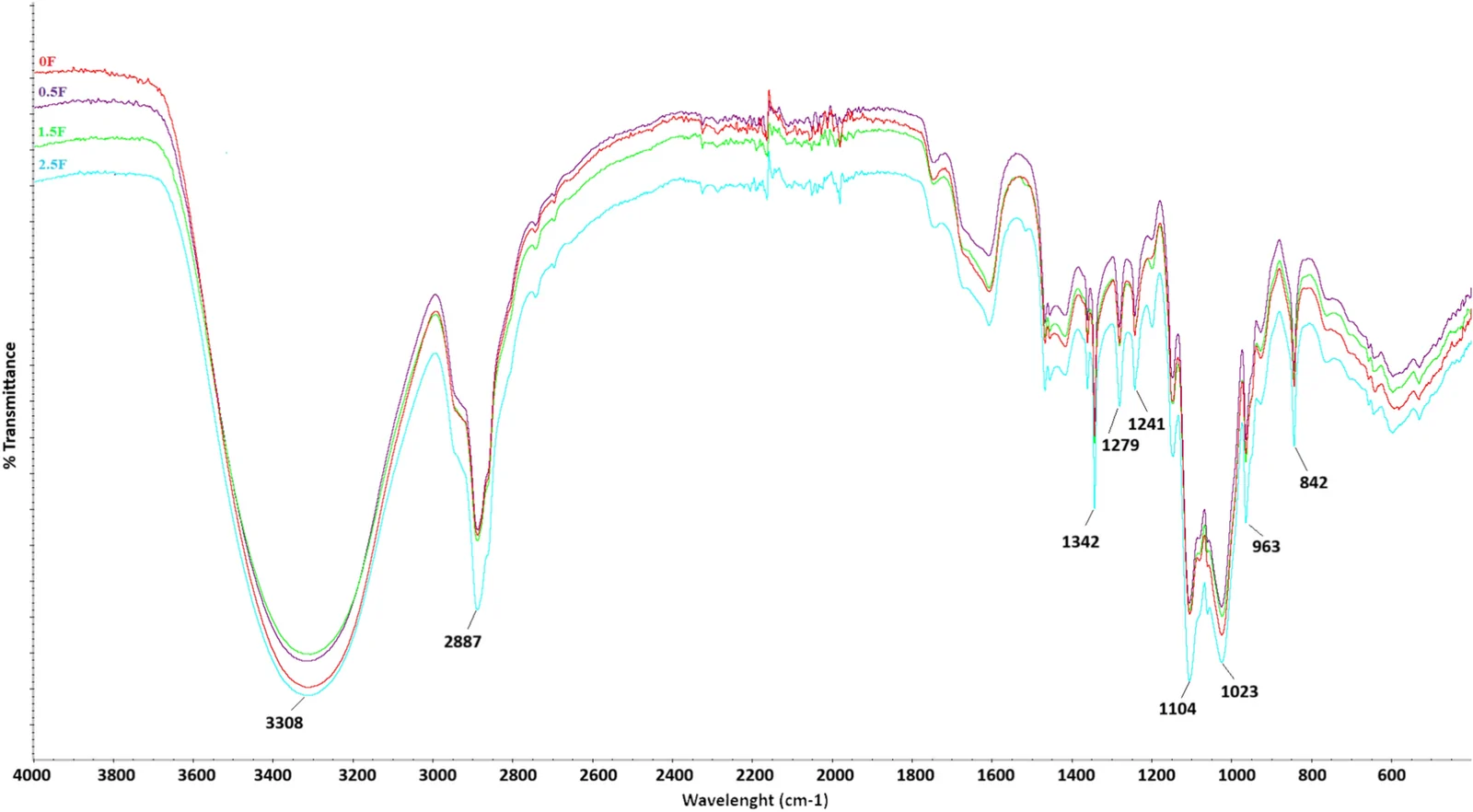
FTIR spectra of bioactive packaging materials.

Figure 5 presents a spectrum with significant peaks at the following wavelengths: 3308 cm^-1^, 2887 cm^-1^, 1341 cm^-1^, 1279 cm^-1^, 1241 cm^-1^, 1103 cm^-1^, 1022 cm^-1^, 962 cm^-1^, 842 cm^-1^. The broad band at 3308 cm^-1^ are attributed to the O–H stretching vibrations of the hydroxyl groups present in the pectin and water molecules in the resulting films^53^. The sharp and long vibrations at 2887 cm^-1^ can be attributed to the C-H stretching vibrations of the groups present in CH_2_ and CH_3_ in pectin^54^. The appearance of these peaks indicates the molecular structure of pectin, which is a plant-derived polysaccharide consisting of a carbohydrate ring^55^. Bands from 1103 cm^-1^, 1022 cm^-1^ and 962 cm^-1^ were attributed to the furanose structure and α- and β-pyranose rings in pectin. The peaks from 1279 cm^-1^, 1241 cm^-1^ and 842 cm^-1^ are associated with C–C and C-O stretching and glycosidic bonds^56^. The spectra obtained confirm the stable structure of the films obtained, which was confirmed by further studies.

### Physical properties of films (density(qs), swelling index (SI), moisture content (MC))

**Fig. 6:**
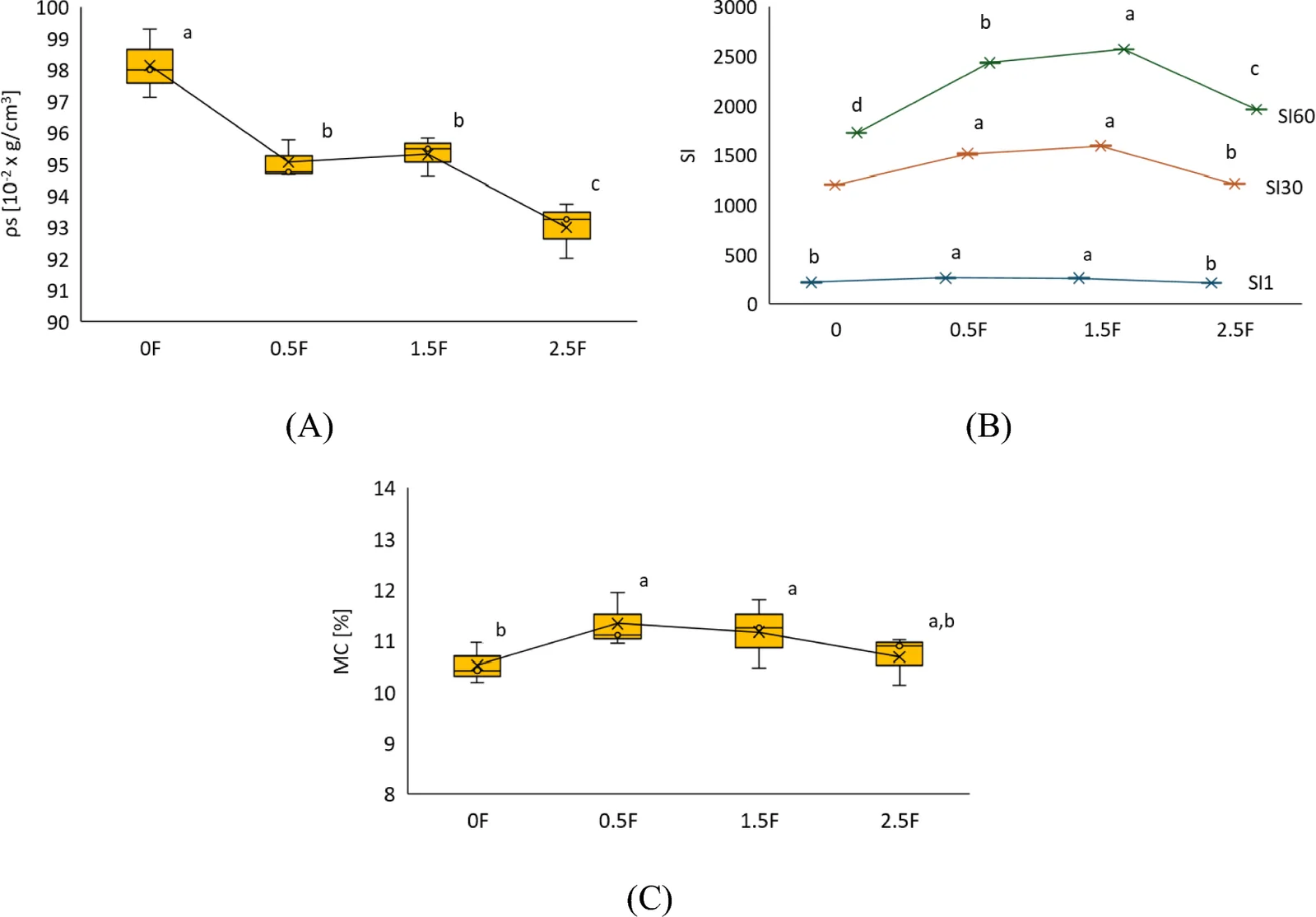
Results of tests for density **(A)**, swelling index (SI) **(B)**, moisture content (MC) **(C)**.

Figure 6 shows the results of density (A), swelling index (B), and moisture content (C) measurements. An increase in the amount of Pau d’Arco extract incorporated into the films led to a slight decrease in film density (p > 0.05). Moisture content (MC) and swelling index (SI) were assessed as key factors determining the water retention capacity of the films^57^. The swelling index reflects the maximum water absorption of a film before structural failure, indicating its potential suitability as a water infiltration material. Swelling behaviour is strongly influenced by the extent of intermolecular interactions within the polymer chains^58^. The swelling index was measured after 1, 30, and 60 min of immersion in distilled water, and it was observed that the addition of the extract increased the SI values. This effect is likely due to enhanced cross-linking between apple pectin, curdlan gum, and polyphenols during film formation. Moisture content and swelling index are closely related to film hydrophobicity, with lower values promoting better physical integrity when in contact with high-moisture foods^59^. Slight increases in MC were observed for the 0.5F and 1.5F samples (p > 0.05), whereas the 0F and 2.5F samples exhibited similar MC values of approximately 10.5%. The decrease in MC in cross-linked films can be attributed to the replacement of water interactions by hydrogen bonding among apple pectin, curdlan gum, and polyphenol-derived polar groups from the Pau d’Arco extract, resulting in reduced water binding^56^.

### Color and opacity analysis of films

**Fig. 7:**
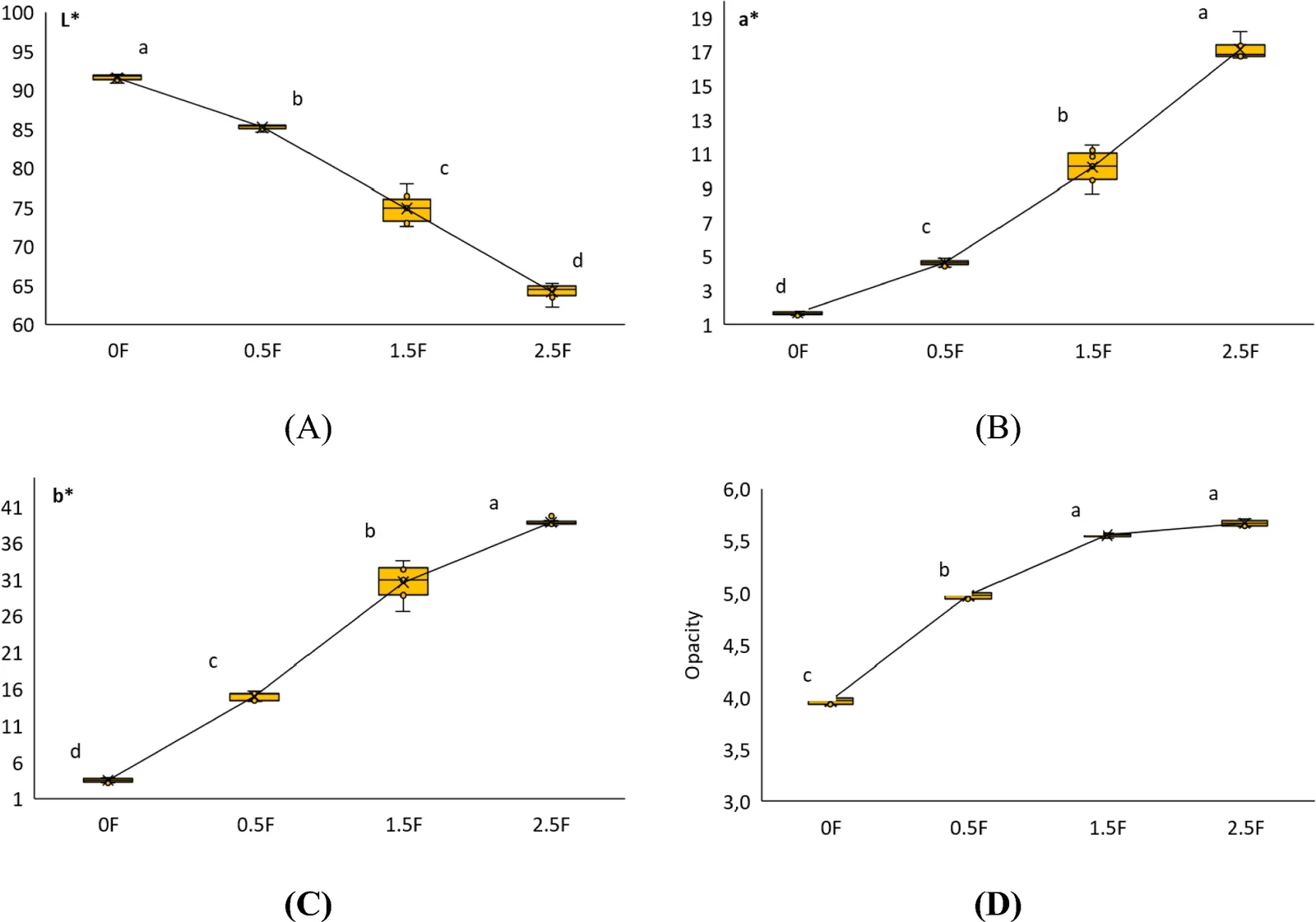
Color test results of the obtained films for each parameter: L* **(A)**, a* **(B)**, b* **(C)** and opacity **(D)**.

Figure 7 presents the results of color measurements of the obtained films. The following parameters were evaluated: L* (lightness), a* (red–green component), b* (yellow–blue component), and total color difference (ΔE). A statistically significant decrease in L* (p < 0.05) was observed with increasing extract concentration, reflecting the dark color of the added Pau d’Arco extract in the polymer matrix. Sample 0F exhibited the highest lightness (L* = 91.61), while the film with the highest extract content had the lowest lightness (L* = 64.20). The values of a* and b* parameters increased with higher extract content. The a* value for sample 0F was 1.69, whereas for sample 2.5F it reached 17.14, indicating a statistically significant increase (p < 0.05) due to the incorporation of the colored extract. Similarly, b* values increased linearly with the extract concentration. These results are consistent with previous studies^60,61^, which reported a decrease in L* and an increase in ΔE after the addition of active substances to pectin matrices. In this study, the ΔE values for the 0.5F, 1.5F, and 2.5F films were 13.49, 32.97, and 47.34, respectively, confirming that the color of the films changed significantly depending on the extract concentration. This effect is attributed to the presence of active, colored compounds in the extract, which, dispersed in the biopolymer matrix, alter the optical properties of the material^62^. The observed color changes were also confirmed by stereoscopic microscopy, which clearly showed the color of the tested samples. Opacity is commonly used to assess the light barrier properties of packaging materials^61^. Incorporation of the extract into the polymer matrix resulted in a significant increase in opacity (p < 0.05), likely due to the formation of cross-links between film components, which increased density and reduced transparency. Opacity also increased proportionally with extract concentration, resulting from the addition of biopolymeric extracts containing polyphenolic compounds, which affect the refractive index and reduce light transmission^63,64^. In this study, higher polyphenol content corresponded to higher opacity. Other studies suggest that increased film opacity may also result from a rougher surface structure, which promotes light scattering^65^. Non-transparent films effectively block light, preventing photo-oxidation of lipids and subsequent product quality deterioration^57,66^.

### Mechanical tests of films

**Fig. 8:**
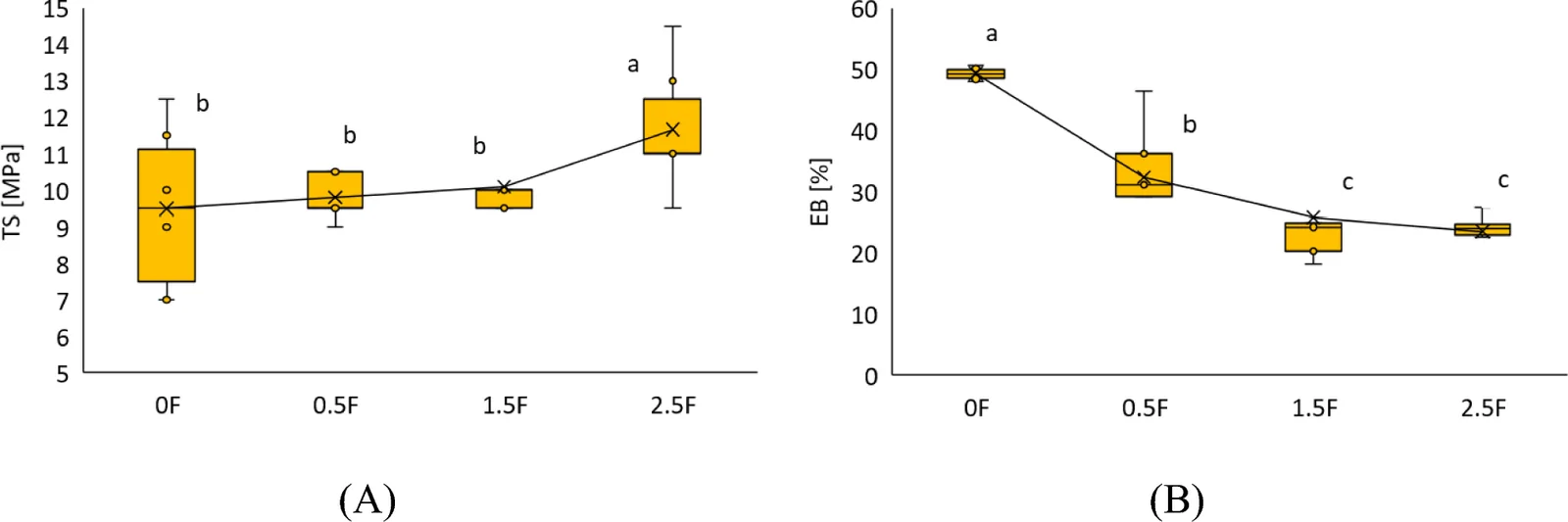
TS **(A)** and EB **(B)** for obtained films.

In testing the developed packaging material, a determination was made of elongation at break (EB, %), which measures ductility, or the ability of the film to stretch before breaking, while tensile strength (TS, MPa) measures the maximum stress the material sample can withstand when stretched before breaking. The tests conducted showed that the TS results for all our samples ranged from 9.50 MPa to 11.67 MPa. A Linear increase in TS with increasing extract concentration was observed. The sample without the addition of extract showed a value of 9.50 MPa and this was higher than the TS value they obtained^61^, where they used films consisting of pectin and CMC. Also, Guo et al.^67^ obtained films based on soy protein isolate (SPI) and apple pectin (PA) where the tensile strength was about 2.52 ± 0.24. In the present study, the highest strength was observed with 2.5 wt.% of extract and was 11.67 MPa. The observed increase in TS due to the addition of Pau d’Arco extract can be attributed to its homogeneous dispersion in the film matrix, which enhanced the film’s cross-linked structure through hydrogen bonding and cross-linking with pectin and curdlan gum molecules^61^. The observed increase in TS caused by the addition of Pau d’Arco extract can be attributed to its homogeneous dispersion in the film matrix, which strengthened the film’s network structure through hydrogen bonding and crosslinking with pectin and curdlan gum molecules^61^. In addition, the higher the polyphenol content of the samples (as confirmed by previous experiments), the higher their strength. This was proven by statistical analysis, which indicated that the mechanical properties of the films are correlated with the sum of polyphenols and flavonoids. Figure 8 shows a plot of tensile strength and elongation at break at the extract concentrations used. This testifies to the strong interaction between PA, CG and Pau d’Arco extract, as well as the well-chosen concentrations. The use of both polysaccharides, as well as the appropriate amount of extract concentrations, did not interfere with the interaction of the polysaccharides, resulting in an increased TS value.

The opposite relationship was observed for the elongation at break of EB. It was observed that as the amount of extractant to the matrix increased, there was a linear decrease in the elasticity of the film. The highest EB value was characterized by sample 0, where EB was 47.67%. This value was higher than the pectin films presented in the paper^68^. Therefore, the addition of curdlan gum affected the elasticity of the obtained samples. For the sample with the highest extract content, i.e. 2.5 wt.%, the EB value was 22.84% (Fig. 8). The inclusion of extract in the matrix significantly (p < 0.05) reduced the flexibility and elasticity of the films. Although a reduction in EB was observed for these films, its value was still higher than the EB value for pectin films, which was presented in the papers^69,70^. Therefore, the present study demonstrated that the effectiveness and mechanical parameters depend on the specific properties of the film-forming components and the preparation technology.

### Barrier properties of films

**Fig. 9:**
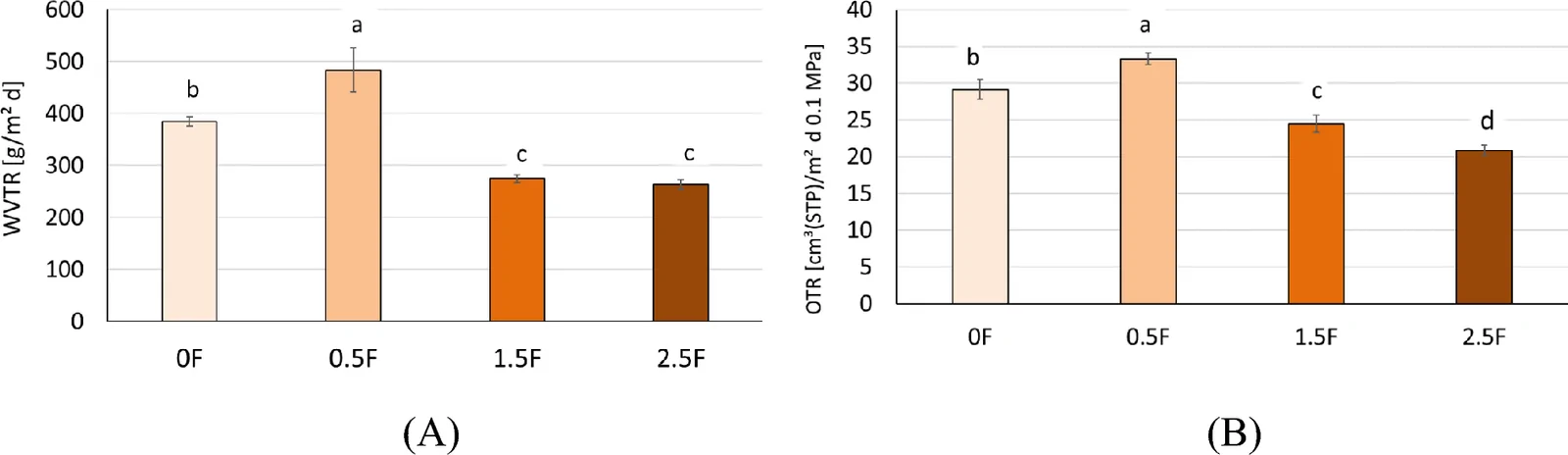
Results of WVTR and OTR tests.

Water vapour and oxygen permeability are key parameters in selecting materials for food packaging and preservation^71^. Figure 9 presents the results of water vapour transmission rate (WVTR) and oxygen transmission rate (OTR) measurements for the films studied. The results showed that increasing the extract concentration enhanced the barrier properties of the films against water vapour and oxygen. The addition of the extract induced statistically significant changes (p < 0.05) in both WVTR and OTR values. The 0F films exhibited barrier values of 384.59 ± 9.01 g/m^2^·d (WVTR) and 29.10 ± 1.35 cm^3^(STP)/m^2^·d·0.1 MPa (OTR). The lowest WVTR and OTR values, corresponding to the highest barrier performance, were observed for the 2.5F sample, with values of 263.19 ± 8.86 g/m^2^·d and 20.86 ± 0.74 cm^3^(STP)/m^2^·d·0.1 MPa, respectively. The increase in permeability observed for the 0.5F sample can be attributed to the dominant plasticizing effect, without significant interactions (hydrogen bonding or van der Waals forces) between the polymer matrix and the polyphenols. In contrast, the reduction in WVTR and OTR values for the other samples is likely due to the formation of a compact, complex structure between apple pectin (PA), curdlan gum (CG), and the polyphenols present in the extract, resulting in a densely packed and tortuous pathway that hinders the permeation of moisture and oxygen. Several factors influence the barrier properties of films, including the microstructure of the polymer network, water sensitivity, degree of crystallinity, and the ratio of hydrophobic to hydrophilic components^72–74^. In the present study, changes in water vapour permeability were associated with the incorporation of the extract, which is rich in polyphenols and flavonoids.

### Migration analysis of films

**Fig. 10:**
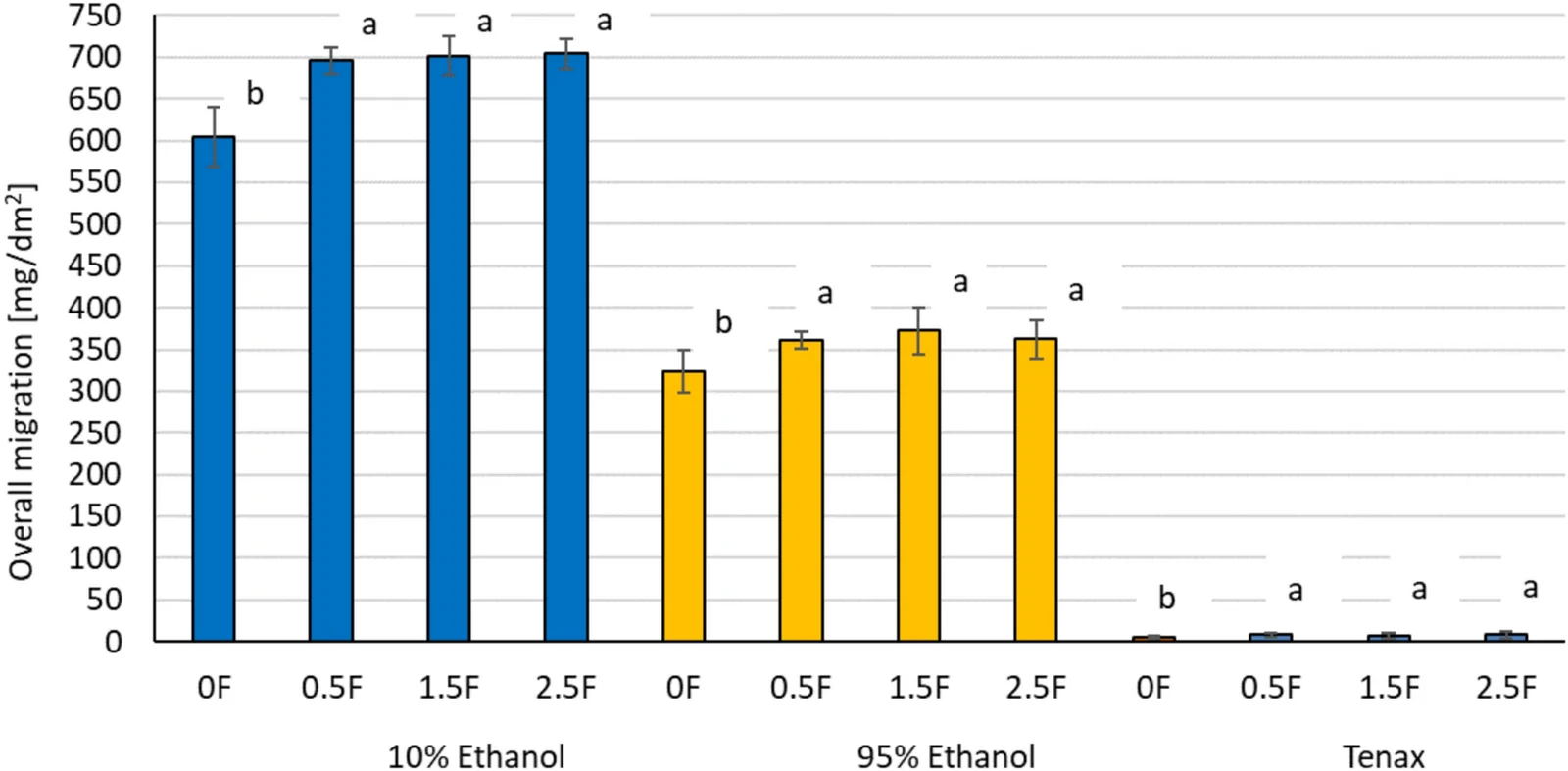
Determination of overall migration into simulant of volatile alternatives for fatty food 10%, 95% ethanol and Tenax at temperature 40 °C for 10 days.

The differences in the amount migrated from the tested films into 10% ethanol, 95% ethanol and Tenax® are most likely due to the polarity of the substances present in the films, their nature and the film structure itself (Fig. 10). The lowest migration occurred in the Tenax® environment (from 5.4 ± 1.8 mg/dm^2^ for the 0F film to 8.2 ± 2.3 mg/dm^2^ for the 0.5F film), as it is a solid sorbent as dry food simulant. Migration levels were much more influenced by the volatility and sorption affinity of the migrated substances in the solid material. For the liquid material, the highest migration levels were determined in 10% ethanol (from 704.3 ± 18.0 mg/dm^2^ for the 2.5F film to 604.3 ± 36.2 mg/dm^2^ for the 0F film). For the migration test in 10% and 95% ethanol, in all cases of the films analysed, more substances were released than in the migration test of the blank sample (film 0F). This indicates the presence in the added extracts of substances with a tendency to migrate into water. This fact was confirmed by the migration results to 95% ethanol, where the migration level was about two times lower than for 10% ethanol. However, at the same time, achieving higher migration values up to 10% ethanol is limited by the total extract content of the film. However, the migration results up to 95% ethanol (from 324.2 ± 25.6 mg/dm^2^ for the 0F film to 372.6 ± 28.0 mg/dm^2^ for the 1.5F film) show that even these results exceed the overall migration limit of 10 mg/dm^2^ specified in Regulation 10/2011. The results also indicate the limited inertness of these materials to liquid food. Higher value of migration into liquid simulants may be caused by hydrolysis or alcoholysis of primary polimers.Therefore, the tested packaging materials may be (based on the migration results) beneficialy used for semi-rigid and rigid types of food (matured meat, meat products—salami etc.) hard cheese etc.

### Images of films

**Fig. 11:**
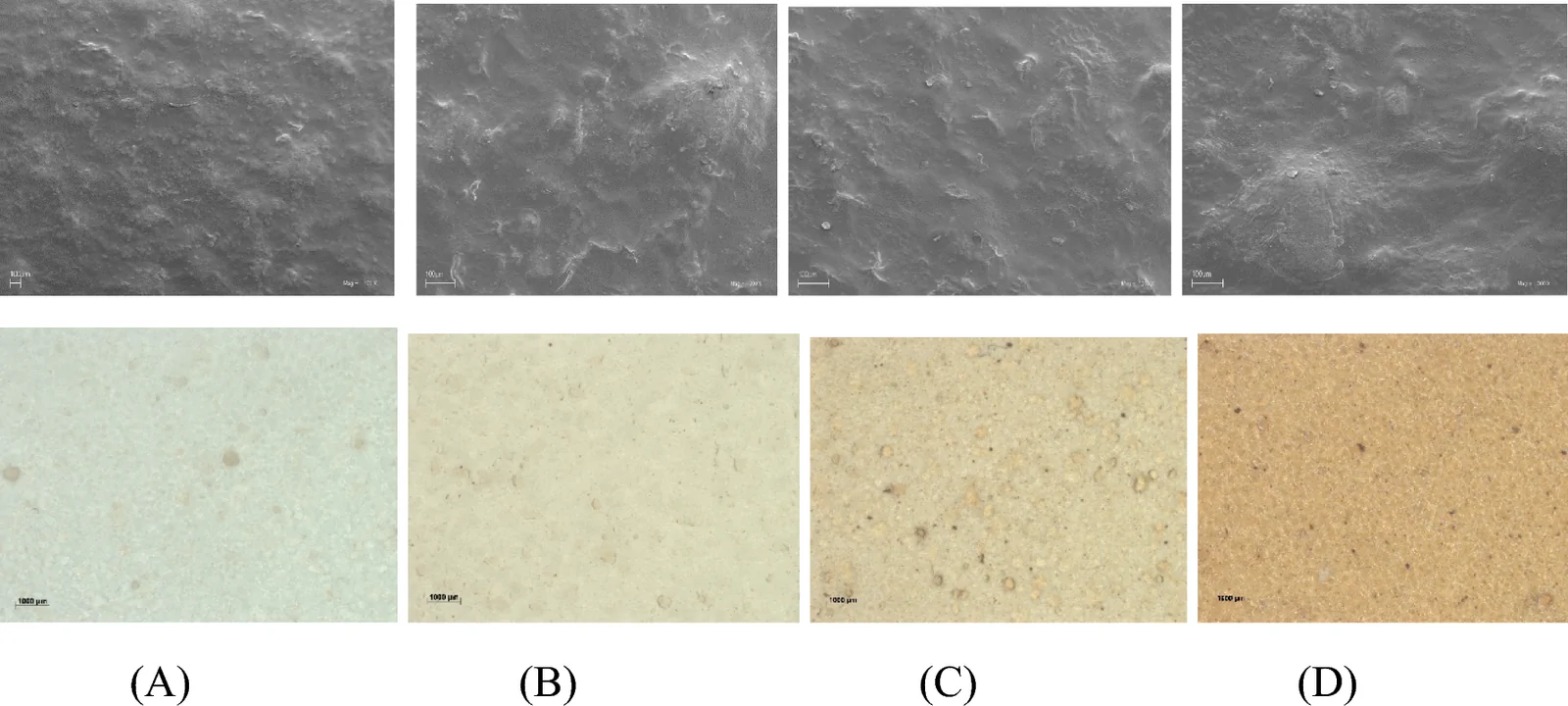
SEM and stereoscopic images for the prepared films (**A**) 0F, (**B**) 0.5F (**C**) 1.5F and (**D**) 2.5F.

The surface of the obtained films was evaluated using scanning electron microscopy (SEM) and stereoscopic microscopy. Some images may show minor surface irregularities; however, detailed analysis confirms that the films possess a homogeneous structure (Fig. 11). SEM observations demonstrated that the combination of both polysaccharides and the addition of the extract resulted in a compact and dense matrix, with no cracks or phase separation, indicating strong and consistent interactions within the polymer structure. Stereoscopic microscopy further confirmed the surface smoothness observed in the SEM images, while also highlighting the expected color changes of the films depending on the extract concentration. The minor irregularities visible in some images are attributed to microscopic features or natural textural nuances of the polymer matrix, which do not affect the overall homogeneity or the mechanical and functional properties of the films.

### Statistical analysis obtained results

**Fig. 12:**
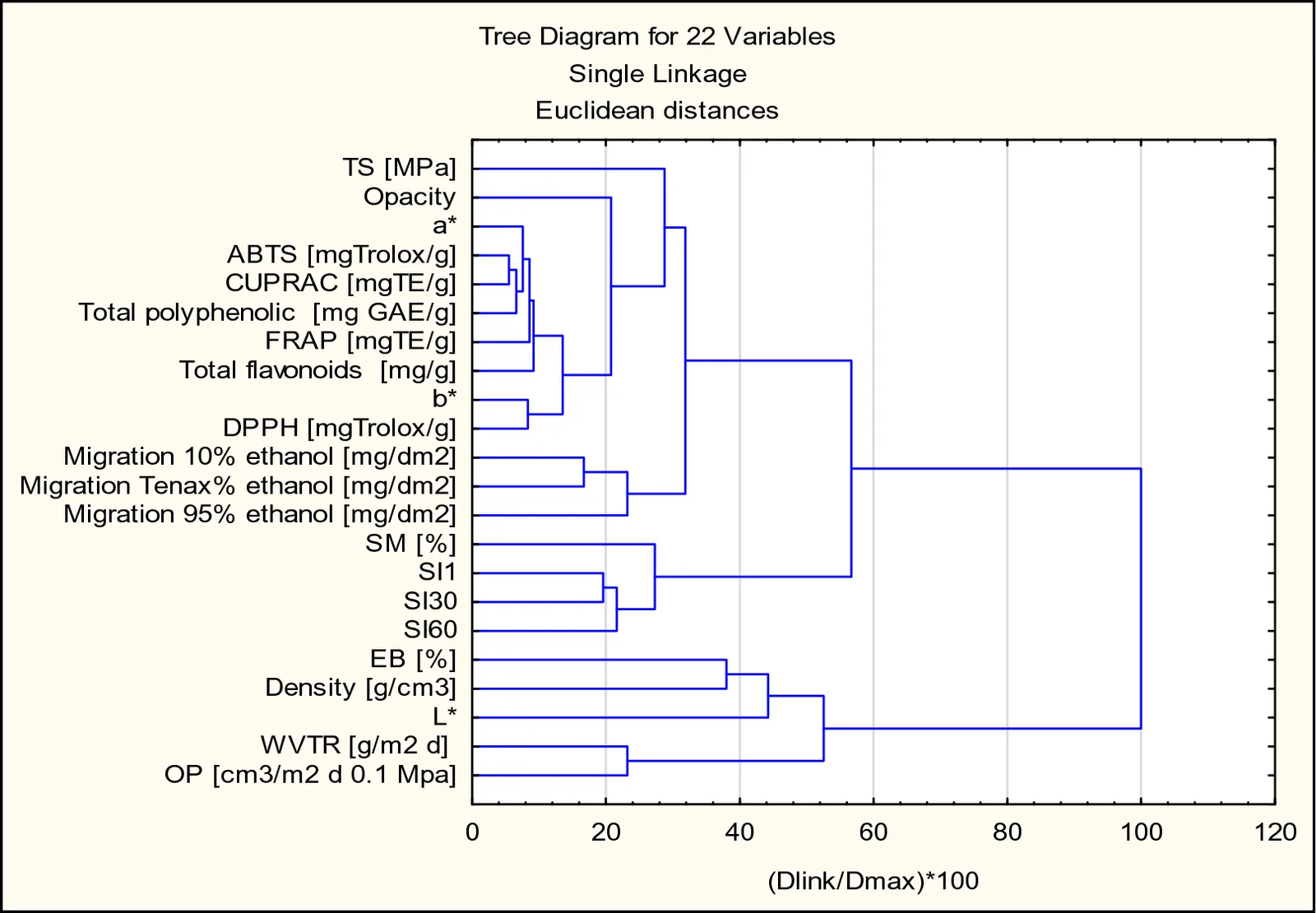
The results of the cluster analysis.

It was observed by statistical analysis that, the parameters of total polyphenols, total flavonoids and antioxidant activity determined by different methods are fully correlated with each other (R = 1). This correlation has already been found in previous studies by many researchers, since polyphenolic compounds exhibit high antioxidant activity. The parameters of antioxidant activity and the total of polyphenols and flavonoids are also fully correlated with the parameters: TS, EB, Migration in 10% ethanol, opacity and L*, a*, b* color parameters. Strongly correlated (0.7 ≤ R < 0.9) were the parameters of OTR and WVTR with TS, EB, Migration in 10% ethanol, opacity, L*,a*,b*, SI1 and parameters of antioxidant activity determined by different methods. To illustrate the structure of all results, cluster analysis was performed. The diagram is shown in Fig. 12.

The cluster analysis showed that antioxidant activity determined by different methods, total polyphenol content, flavonoid totals are most closely related, and these parameters form the least distant relationships. All migration parameters are related in a close way. The analysis also showed that WVTR and OTR, EB and density are related and form direct and least far-ranging correlations, and swelling coefficients at 1, 30 and 60 min. are related to moisture content. These correlations are not necessarily reflected in the values of rank correlation coefficients, since the R-score for ranks characterizes only linear relationships, while the effects studied may be higher degree relationships.

## Conclusion

In this study, bioactive polysaccharide-based films were successfully developed using apple pectin and curdlan gum as the polymer matrix, with Pau d’Arco extract incorporated as a functional additive. The addition of Pau d’Arco extract imparted significant antioxidant properties to the films, with polyphenol and flavonoid contents fully correlating with antioxidant activity. The films exhibited concentration-dependent changes in color, mechanical strength, and barrier properties, highlighting the multifunctional role of Pau d’Arco extract within the polymer matrix. These findings demonstrate the high potential of the developed films as sustainable, active packaging materials, capable of prolonging the shelf life of packaged foods while reducing reliance on synthetic additives. The study provides a promising approach for designing environmentally friendly, bioactive packaging systems suitable for food industry applications.

## Data Availability

All data generated or analysed during this study are included in this published article.
